# Complete Genome Analysis of Campylobacter jejuni subsp. *jejuni* Isolated from Bloodstream Infection

**DOI:** 10.1128/MRA.00065-21

**Published:** 2021-03-18

**Authors:** Jaya Lakshmi SS, Dhiviya Prabaa Muthuirulandi Sethuvel, Ashtawarthani Baskaran, Balaji Veeraraghavan

**Affiliations:** a Department of Clinical Microbiology, Christian Medical College, Vellore, India; University of Maryland School of Medicine

## Abstract

We report here the complete genome analysis of a clinical Campylobacter jejuni strain sequenced by a hybrid assembly approach. A hybrid assembly approach provided a complete genome sequence of C. jejuni that contains a 1,681,375-bp chromosome and 47,467-bp plasmid carrying various virulence and antimicrobial resistance determinants.

## ANNOUNCEMENT

*Campylobacter* spp. are one of the common causes of bacterial gastroenteritis in humans, with Campylobacter jejuni being the commonest (1–3). Bacteremia, another complication of C. jejuni infection, was estimated to occur in ∼1% of the cases with appreciable mortality rates (4). Although campylobacteriosis is usually self-limiting, antimicrobial treatment is recommended for severe cases (5). Fluoroquinolones and macrolides are the current drugs of choice. Here, we describe the complete genome analysis of Campylobacter jejuni isolated from bloodstream infection.

C. jejuni subsp. *jejuni* (strain BA18682) isolated from a blood sample received for routine bacteriology at the Department of Clinical Microbiology, Christian Medical College, Vellore, India, was studied. Genomic DNA was purified from Campy blood agar plate (BAP) medium after 48 hours of incubation at 42°C under microaerophilic conditions using the QIAamp DNA minikit (Qiagen, Hilden, Germany). Genomic DNA quality and quantity were assessed using Nanodrop spectrophotometry (ThermoFisher, USA) and Qubit 3.0 (ThermoFisher, USA), respectively. Library preparation for short-read sequencing was performed using the Ion Plus fragment library kit (Life Technologies, Carlsbad, CA) with 400-bp read chemistry using an IonTorrent personal genome machine (PGM) (Life Technologies) as per the manufacturer’s instructions.

Similarly, a long-read DNA library was prepared using the SQK-LSK108 ligation sequencing kit (v.R9) along with the EXP-NBD103 native barcode expansion kit following the manufacturer’s protocol (Oxford Nanopore Technologies, Oxford, UK). Sequencing was performed with FLO-MIN106 R9 MinION flow cells according to the manufacturer’s protocol. The Fast5 files generated were base called with Albacore v.2.0.1 (https://nanoporetech.com/about-us/news/new-basecaller-now-performs-raw-basecalling-improved-sequencing-accuracy). Long reads were error corrected with Canu v.1.7 using the “-correct -nanopore-raw” module (6). The quality of the MinION reads was assessed using MinIONQC (https://github.com/roblanf/minion_qc). The hybrid assembly was performed using the resulting Ion Torrent reads (1,571,892) and MinION reads (1,745,808) using Unicycler (v.0.4.7) as described earlier (7–9). Unicycler utilizes SPAdes v.3.12 by default to assemble the short reads along with error correction and quality checks. Consequently, it trims and generates the short-read assembly graph (10). The reads were polished with multiple rounds of Pilon (v.1.22) to reduce the base-level errors as described previously (11). Default parameters were used for all software.

**FIG 1:**
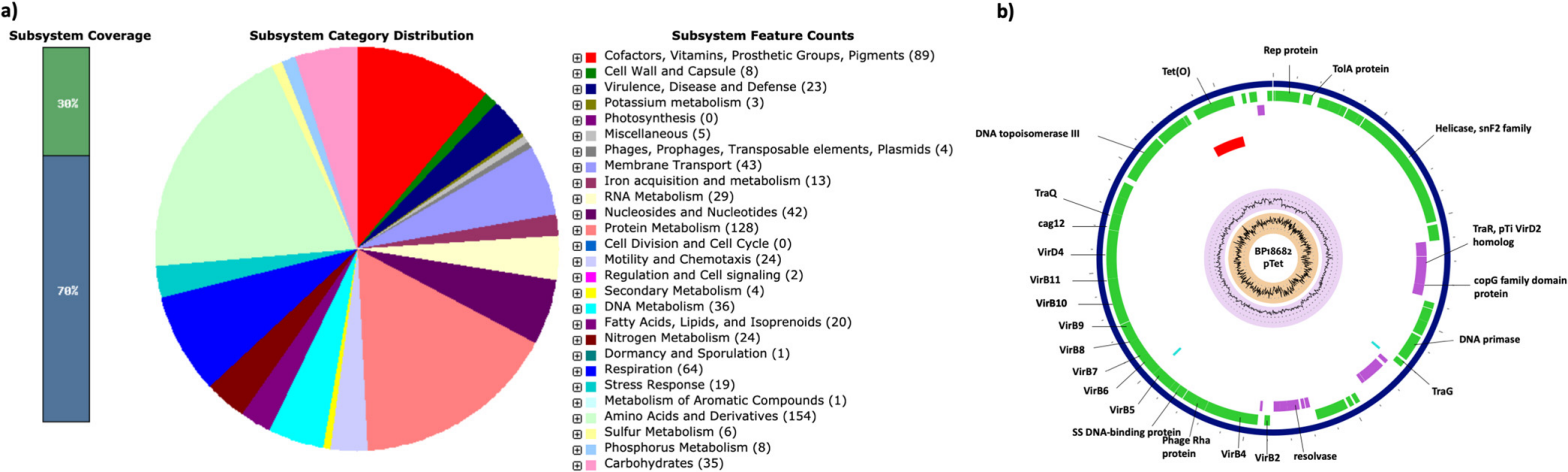
(a) Subsystem categories of C. jejuni annotated by RAST server. Bar diagram on the left shows the subsystem coverage. The pie diagram shows the distribution of subsystem features. (b) Circular map of pTet plasmid (BA18682) showing only genes with known functions.

The genome was annotated using the NCBI Prokaryotic Genome Annotation Pipeline (PGAP). The genomic features of the isolate are given in Table 1. A hybrid assembly provided a complete genome of C. jejuni that contains a 1,681,375-bp chromosome and 47,467-bp plasmid. The distribution of subsystems in C. jejuni annotated by RAST server-based annotation and the circular representation of plasmid (pTet) identified are shown in Fig. 1. Several genes associated with invasion, adherence, motility, polysaccharide capsule, and lipooligosaccharide, including toxin-related genes, were identified in the chromosome using the VFDB database (12). Genes involved in the type IV secretion system (T4SS) were identified in the plasmid (pTet). Antimicrobial resistance determinants for beta-lactams (*bla*_OXA-450_), tetracycline [*tet*(O)], and fluoroquinolones (*gyrA*, T86I) were identified through the standalone BacWGSTdb database with default parameters (13). MLST analysis revealed that the isolate belongs to sequence type 10023 (ST10023). No mobile genetic elements were identified except an incomplete prophage region.

**TABLE 1:** Genomic data obtained from Campylobacter jejuni sequenced in this study

Genomic feature	Value
Total length (bp)	1,806,568
Coverage (×)	31.4
No. of contigs	67
*N*_50_ value (bp)	164,739
Total no. of genes	1,791
Total no. of CDS^*a*^	1,735
Total no. of pseudogenes	168
No. of tRNAs	44
No. of rRNAs	3
GC content (%)	33
No. of subsystems	195
No. of virulence factors (VFDB)	102
No. of resistance factors (CARD)	8

a CDS, coding DNA sequences.

### Data availability.

The complete genome sequence of C. jejuni has been deposited in GenBank under accession numbers CP050683 (chromosome) and CP050684 (plasmid, p18682). The raw reads from Ion Torrent and MinION sequencing are available in the NCBI Sequence Read Archive (SRA) under accession numbers SRX9767595 and SRX9772619, respectively. The BioProject accession number is PRJNA615343, and the BioSample accession number is SAMN14451476. RAST annotation files are available online at Figshare (https://doi.org/10.6084/m9.figshare.13941860).
